# Dietary Choices of New Zealand Women during Pregnancy and Lactation

**DOI:** 10.3390/nu12092692

**Published:** 2020-09-03

**Authors:** Kimberley Brown, Pamela von Hurst, Jeanette Rapson, Cathryn Conlon

**Affiliations:** School of Sport, Exercise and Nutrition, College of Health, Massey University, Auckland 0745, New Zealand; k.brown1@massey.ac.nz (K.B.); P.R.vonHurst@massey.ac.nz (P.v.H.); J.Rapson@massey.ac.nz (J.R.)

**Keywords:** New Zealand, pregnancy, lactation, dietary choices, food safety, information sources

## Abstract

Dietary recommendations during pregnancy and lactation have become increasingly complex, and sources of information more numerous but not always reliable, potentially causing confusion and unsafe choices. Women were recruited during pregnancy or within six months postpartum and completed questionnaires on dietary choices, food safety, and sources of nutrition information. Women (*n* = 458) from around New Zealand participated in the study. They consumed a wide range of foods and beverages and reported various dietary changes. In pregnancy, women commonly avoided alcohol (92%), raw milk products (86%), and raw, smoked, or pre-cooked seafood and fish (84%), and made changes due to food safety concerns. Influential advice was acquired from a range of sources including midwives (37%) and the New Zealand pregnancy and breastfeeding guidelines (25%) during pregnancy. Food avoidance was less common in lactation. However, fewer women consumed milk products during lactation (64%) than pregnancy (93%). Potentially unreliable sources were used more frequently in lactation including alternative health practitioners (26%) and family or friends (12%), and dietary changes were often made in response to infant symptoms without supporting evidence. This study highlighted a need for good communication of evidence-based recommendations to women, especially during lactation.

## 1. Introduction

Health and nutrition concerns of women often become more prominent during pregnancy and lactation [1]. Women frequently report changing their diet to achieve the best possible fetal and maternal outcomes [2,3,4,5]. Evidence-based guidelines in NZ “Food and Nutrition Guidelines for Healthy Pregnant and Breastfeeding women” (NZPBG) developed by the Ministry of Health in 2006 promote food and lifestyle-based recommendations [5]. Food-based recommendations change to meet altered nutrient requirements, particularly those for iron, calcium, vitamin B12, iodine, and folate, during pregnancy and lactation [5]. As women eat food, rather than nutrients, recommendations advise the selection of foods within the four food groups and women are recommended to choose additional servings of vegetables; dairy products; lean meat, poultry, seafood, eggs, nuts, seeds, or legumes; and breads and cereals [5]. Currently there is a considerable amount of literature available surrounding the nutritional requirements of women during pregnancy and lactation and it is widely accepted that favourable dietary choices are important to achieve optimal outcomes for both women and their offspring [6,7,8]. Despite the importance of women’s dietary choices to meet their altered nutritional requirements, there is a lack of evidence to suggest what choices women make. This is particularly true during lactation in NZ. Dietary choices, which are defined by the European Food Information Council as the complex decisions individuals make when choosing and consuming food and beverages, provide insight into what foods and beverages women consume, limit, and avoid during pregnancy and lactation [9]. Such information is important for health professionals, to ensure women receive relevant and appropriate dietary education to support the best possible outcomes for women and their offspring [10,11].

There is a wealth of food safety advice for pregnant women because of the increased risk of foodborne illness [12,13,14,15]. Food safety recommendations are therefore particularly complex and comprehensive [5]. Women have an increased susceptibility to listeriosis, toxoplasmosis, salmonella, botulism, influenza, varicella, and methyl mercury toxicity during pregnancy [12,13,14,15]. They also have an increased risk of infection severity, miscarriage, premature birth, still-birth, and fetal and maternal mortality [12,14,16,17]. High-risk foods that have been identified include soft cheeses, cold deli salads, cold cooked or smoked meats, processed meats, raw products, soft-serve ice cream, tahini, and ready-to-eat meals [5,17,18].

Evidence from NZ and elsewhere suggests that women are likely to avoid a variety of foods during their pregnancy based on the advice they receive from health professionals [4,19,20]. Whether women who are breastfeeding continue to avoid a variety of foods is less well understood. Dietary advice during pregnancy and lactation is important to support the recommended dietary changes to optimise nutritional intake during this period [5]. Previous studies have identified midwives, general practitioners (GP), printed media, friends, and family as common nutrition information sources [21]. This study aimed to determine women’s dietary choices, food safety practices, and sources of nutrition information during pregnancy and lactation. This evidence is important to understand what support women require to make optimal dietary choices during pregnancy and lactation.

## 2. Materials and Methods

This cross-sectional, observational study recruited pregnant and lactating women throughout NZ. To meet the inclusion criteria women were required to be pregnant or within six months postpartum and breastfeeding. There were no exclusion criteria. Recruitment was conducted between January and June 2019 via social media, professional associations, posters, word of mouth, and personal contact. Participants were self-selected and therefore not representative of the NZ population. The questionnaires were developed by the research team of registered nutritionists and dietitians to align with the current NZPBG [5]. Questionnaires from the Growing Up in NZ study (GUiNZ) provided some guidance for question flow and wording [22,23]. Dietary recommendations from the Ministry of Primary Industries and the NZPBG guided foods that were included in the questionnaires. Foods that are commonly recommended to pregnant and lactating women were included to provide insight if women’s food choices reflect recommendations [5,24]. Pilot testing was conducted with fourteen pregnant or breastfeeding women. Pilot testing resulted in wording changes, clarification of instructions, and minor changes in the order of questions to increase the readability of the questionnaire.

Demographic information included health status, parity, food security, age, ethnicity, qualifications, and geographical location. Food choice questionnaires (FCQ) explored: avoidance, addition, and limitation of foods; supplement use; and information sources. The Food Frequency Questionnaires (FFQ) explored women’s daily, weekly, monthly, and occasional consumption of fruit; vegetables; lean meat, poultry, seafood, eggs, nuts, seeds or legumes; dairy products; breads and cereals; and beverages. As our research aimed to investigate women’s dietary choices, and nutrient assessment was not intended, portion sizes were not included in questionnaires.

The questionnaires took approximately 10–15 min to complete. Questionnaires favoured closed questions to maximise responses. Open questions were used to allow women to express additional information that they felt was appropriate. This information has been used to provide further explanation for statements made where appropriate. The option of “choose not to answer” was provided to skip questions.

Ethical approval to conduct this study was gained from the Massey University Human Ethics Committee (MUHEC): Southern A (application 28/09).

Statistical analysis was conducted using IBM SPSS statistics (version 24.0, IBM Corp, Armonk, NY, USA). Descriptive statistics, including mean, standard deviation, frequency, and percentage were calculated through multiple response sets and custom tables. Chi-squared tests were used to compare women’s education level and alcohol intake. The homogeneity of the sample demographics did not allow an analysis of factors influencing dietary intake.

## 3. Results

**Figure 1:**
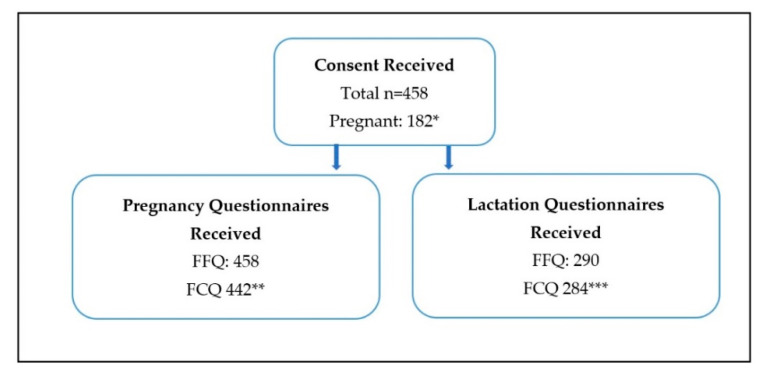
Questionnaires completed. * of women who were pregnant, 14 gave birth and accepted an invitation to complete the lactation questionnaires within recruitment time. ** The pregnancy FCQ was not completed by 16 women. *** The lactation FCQ was not completed by 6 women.

Women who completed the pregnancy FFQ were included in the final analysis (*n* = 458) (Figure 1). The pregnancy FCQ was completed by 442 of these women. From the study population 182 women completed the pregnancy questionnaires during their pregnancy and 276 completed them retrospectively. Eligible women (*n* = 290) then completed the lactation FFQ (*n* = 290) and FCQ (*n* = 284). Respondents were largely well-educated NZ European women with adequate food availability (96%) from all around NZ (Table 1). Women mostly reported good health before pregnancy (94%). Thirty-one percent of women reported a change in their health during pregnancy, the majority (83%) felt that their health status declined. Iron deficiency and heartburn were the most common diagnoses (Table 1).

### 3.1. Dietary Choices

Women consumed a range of foods during pregnancy and lactation (Table 2). They frequently added or increased foods to their diet in pregnancy (69%) and lactation (53%) (Table 3). Most commonly, dairy products, nuts, and green leafy vegetables were added. Reasons for adding or increasing foods were to increase dietary iron and/or calcium (52%), meet food cravings (49%), and/or support their baby’s health (46%). During lactation, the most common reasons were to support baby’s health (42%), increase dietary calcium and/or iron (36%), and/or meet food cravings (29%). In pregnancy (88%) and lactation (70%), women reported avoiding foods, including alcohol, raw milk and milk products, raw, smoked, or pre-cooked fish or seafood (Table 4). The main reason for avoidance during both pregnancy (88%) and lactation (48%) was following the NZPBG. Other reasons in pregnancy were advice from health professionals (68%), advice from the internet, magazines, books, newspapers (36%), and advice from family or friends (27%). During lactation, avoidance reasons included advice from health professionals (28%), food preferences (26%), and food safety concerns (12%). Food limitation was more common during pregnancy (81%) than lactation (66%) (Table 5). Women limited food in pregnancy because of mercury concerns (35%) and food preferences (35%). In lactation, dislike of foods (25%) and mercury concerns (7%) were the most common reasons.

Women’s dietary choices were affected by a variety of factors. In pregnancy women frequently commented that nausea, previous pregnancy complications, or in vitro fertilisation.

IVF pregnancies influenced food choices. Diets were more relaxed when suffering from nausea and were stricter if conception was difficult.

“*First trimester nausea caused a big change in my food choices—fewer veggies and more salty carbs*.”

“*This pregnancy is a result of IVF after years of infertility. Anxiety related to this may have caused me to be even more cautious than I might have been otherwise*.”

Common influences during lactation included tiredness, inability to prepare food due to limited time, and baby’s symptoms.

“*Time to eat well is very difficult. Getting a variety of foods each day is so hard with a new-born!*”

“*The biggest change in my diet is that I am eating way more store-bought products rather than making them myself (e.g., muesli bars, bliss balls, muesli, sauces, nut butter, etc) this is mainly to save time and energy*”.

#### 3.1.1. Milk and Milk Products

Daily consumption of cow’s milk was more commonly reported in pregnancy than lactation (Table 2). Lactating women reported avoiding milk products because of a belief that dairy caused infant colic, reflux, or allergic symptoms, or following advice from health professionals, family or friends, or information sourced from the internet.

“*Have now cut out dairy from diet for baby not confirmed issue but precaution due to reflux and colic*”.

“*Trying to be dairy-free for my bubs*”.

Women who chose milk alternatives also more frequently reported choosing non-fortified milk alternatives (52% in pregnancy and 56% lactation).

#### 3.1.2. Lean Meat, Poultry, Seafood, Eggs, Nuts, Seeds, or Legumes

A range of lean meat, poultry, seafood, eggs, nuts, seeds, and legumes were consumed (Table 2). Some women who followed meat-free diets commented that they had added sources of animal meat to their diet during pregnancy.

“*Prior to being pregnant I was vegan, I am no longer vegan and will go back to being vegan once I am done breastfeeding*”.

“*Pre-pregnancy I mainly ate a vegetarian (pescatarian) diet as my husband is a vegetarian. I have been trying to increase my consumption of meat since becoming pregnant*”.

Oily fish (salmon, tuna, mackerel, and sardines) was consumed at least once a week by a third of both groups. Women frequently commented that they increased their fish intake, however, only 13% reported increasing their intake of salmon (Table 3).

“*I spent the first trimester vegan but then relaxed and became vegetarian. Over the last month (roughly) I began to eat a small amount of fish*”.

#### 3.1.3. Fruit, Vegetables, Breads and Cereals, and Beverages

Both groups consumed a wide range of fruit, vegetables, breads and cereals, and beverages (Table 2). Women often reported their fruit and vegetable choices were influenced by seasonality. Women most frequently consumed an average of three types of fruit and three types of vegetables daily in pregnancy. In lactation, women reported consuming an average of two types of fruit and five types of vegetables daily. Both pregnant (82%) and lactating (84%) women consumed bread daily. Wholegrain versions were chosen by 29% pregnant and 39% of lactating women. Caffeine-containing beverages were consumed daily by approximately half of women in both groups (Table 2). Women were aware of caffeine recommendations and commonly reported avoiding caffeine entirely or choosing decaf versions rather than limiting intake.

“*I have drunk decaf tea and coffee all pregnancy*”.

Alcohol was the most commonly avoided beverage during both pregnancy (92%) and lactation (62%). When categorized by education level, tertiary-educated women were two times more likely to avoid alcohol.

#### 3.1.4. Supplements

During pregnancy 96% of women took folic acid supplements, 95% took iodine supplements, and 70% took other supplements including iron, calcium, magnesium, fish oil, selenium, zinc, vitamin C, vitamin B complex, probiotics, and vitamin D. During lactation, 26% continued taking folic acid supplements and 63% continued iodine supplements. Other supplements (60%) included garlic oil, fenugreek, Chinese herbs, evening primrose oil, spirulina, blessed thistle, brewer’s yeast, selenium, collagen, and glucosamine. Various reasons for supplement use were reported (Table 6).

### 3.2. Food Safety Practices

Women reported being aware of food safety recommendations and 68% purposely avoided food and beverages during pregnancy because of food safety concerns (Table 4). Additionally, 88% reported using NZPBG, which incorporates food safety recommendations. Some women (11%) did not avoid any foods or beverages. Women commented being more relaxed about food safety and dietary choices during consecutive pregnancies because of limited time with multiple children, fatigue, or finding recommendations hard to adhere to.

“*I tried to eat as healthy as possible, the national food guidelines were helpful, I was surprised by the amount of food restrictions due to listeria risk*”.

“*Never ate anything considered to be a ‘risk’ food for pregnant women*”.

### 3.3. Information Sources

Women’s most influential information source varied between the groups (Table 7). Information sources such as midwives and NZPBG were more frequently used in pregnancy than what was observed in lactation. In lactation the use of alternative health practitioners was frequently reported.

Information about food and beverage avoidance was more commonly reported than what foods and beverages to consume in pregnancy (Table 8). In lactation, there were no clear trends.

The majority of women chose midwives to be their LMC (92%), with 6% choosing an obstetrician, and 2% having shared care due to twin pregnancies. Dietary advice from LMCs was received by 87% of women. Information received tended to focus on what not to do (Table 8). Antenatal classes were attended by 39% of women during pregnancy, of whom 40% received dietary advice. Multiparous women reported attending antenatal classes in previous pregnancies which was why they did not attend classes during their current pregnancy. Other reasons for not attending classes were limited availability and timing. Many were scheduled to start classes in the approaching weeks. Women used a range of information sheets during pregnancy. Food safety (54%) and general healthy eating (40%) brochures were more commonly used by women than alcohol-related (13%) brochures.

Lactating women reported the use of many potentially unreliable information sources (Table 9).

## 4. Discussion

Women, in this geographically diverse, predominantly NZ European cohort, chose a variety of foods and beverages during pregnancy and lactation. The majority of participants were well-educated, of good health and food secure, therefore this cohort is not representative of the NZ population. It does, however, represent women who are educated and food secure with potentially fewer barriers to making optimal dietary choices [19,25].

### 4.1. Foods Added or Increased, Limited, and Removed

Food addition/increase, limitation, and removal trends in pregnancy were similar to those observed in GUiNZ which reported 41% added and 87% avoided foods during pregnancy [26]. This was not dissimilar to the 48% who added, and 92% who avoided, one or more foods or beverages in this present study. Food limitation has not been reported from the GUiNZ pregnant cohort, therefore this study provides new information that approximately 80% of women also limit their consumption of certain foods in pregnancy [26]. This study also provides valuable information on foods being consumed during lactation, finding that 50% of women did not add or increase foods, 70% avoided foods and/or beverages, and 66% limited foods. During lactation there are no recommendations for food and beverage avoidance, women are, however, recommended to limit their alcohol and caffeine intake [5]. Considering the large proportion of lactating women who avoid and/or limit foods and beverages within this study, there is evidence to suggest women’s dietary choices are influenced by sources beyond the current NZPBG recommendations. The reason for this is unclear; however, communication of dietary recommendations and confusing messages have been previously identified in NZ’s health information for pregnancy, suggesting possible reasons for women not adhering to dietary recommendations during lactation [26]. Further research is required to distinguish why women avoid and limit foods in lactation.

### 4.2. Reasons for Dietary Change

Women reported a variety of reasons for dietary changes including following the NZPBG, to increase dietary iron/calcium, food cravings, food safety, to support babies’ health, and because of advice from health professionals. Dietary choices were also impacted by factors such as morning sickness and conception difficulty in pregnancy and fatigue and infant symptoms in lactation. A large proportion of women reported using the NZPBG when making dietary changes, justifying their importance. There are limitations to the current NZPBG as they do not provide comprehensive recommendations for managing and making dietary changes, how to cope with fatigue when preparing meals, and how the maternal diet affects infant symptoms [5]. Additionally, there is a lack of emphasis on what women can eat during pregnancy and lactation, which could account for more women removing and limiting foods than adding.

### 4.3. Milk and Milk Product Consumption

Milk consumption was less commonly reported in lactation as women often feared they were causing infant discomfort. Women’s consumption of calcium-fortified milk alternatives was also low, with over 50% of chosen milk alternatives being unfortified. Removing calcium sources, such as milk, without replacing with fortified alternatives increases a woman’s risk of not meeting calcium recommendations during lactation [5]. In NZ milk is the highest dietary calcium source (27%) for all age groups and genders [27].

There is evidence to suggest that maternal restriction of antigen foods, such as cow’s milk and eggs, is beneficial for some infants suffering from atopic eczema [28]. The evidence for the protection from other allergies is weak, which supports the NZPBG recommendations that women should not purposely avoid foods [28]. Food avoidances could result in dietary deficiencies and increase the infant’s risk of developing allergies [5,28,29]. Physiological changes, especially bone turnover, are protective of the infant’s average procurement of 260 mg/day of calcium at the mother’s expense [30,31]. Women’s bone mineral density (BMD) has been shown to decrease by 1–3% per month during lactation, exceeding postmenopausal loss of 1–3% per year [30]. Bone density is then regained once breastfeeding is ended and menstruation returns [31]. Current research suggests that bone loss is independent of maternal calcium intake and low-calcium diets during lactation do not increase a woman’s risk of osteoporosis in later life [5,32]. BMD is, however, affected by breast milk output, which explains why women with multiple offspring have an increased bone mineral loss [33]. Exceptions have been observed for adolescents who also require calcium for skeletal growth or those with higher calcium requirements including multiple fetuses or those undergoing heparin treatment [34,35]. There is a lack of evidence about the long-term consequences of low-calcium diets in lactation and the implications if low-calcium diets are continued after lactation [33,36,37,38,39,40]. Because a significant proportion of women are avoiding calcium-rich foods during lactation it is possible that the current calcium recommendations of 1000 mg/day are not being met [5]. Additionally, there is a significant amount of confusion surrounding the maternal diet and infant symptoms suggesting the need for further recommendations surrounding the benefits and consequences of food avoidance in lactation.

### 4.4. Supplements

Routine multivitamin supplementation is not normally required during pregnancy and breastfeeding if an adequate diet is consumed [5]. The NZPBG, however, do recommend that woman take a folic acid supplement during the first 12 weeks of pregnancy, secondary to high requirements and the difficulty to meet recommendations from food alone [5]. Additionally, since the NZPBG were developed subsequent recommendations have emerged for iodine supplementation in pregnancy and lactation [41]. Supplement intake was higher than previously observed in NZ, with over 95% of women taking folic acid and iodine during pregnancy in this cohort. In 2018, 84% of 535 NZ women adhered to both folic acid and iodine intake during pregnancy [42]. In the same study, fewer women took iodine in lactation (63%). Iodine supplementation in pregnancy and lactation was higher than identified in a 2011 study (70% and 36%, respectively) that took place soon after supplements were recommended, suggesting that more women are aware of iodine recommendations [43]. A significant number of women took alternative supplements in both pregnancy (70%) and lactation (60%) because of advice from health professionals or following the NZPBG. There are currently no guidelines supporting the use of these supplements, suggesting that women require further evidence-based recommendations about alternative supplements [5]. Of the alternative supplements reported, there is limited evidence to support beneficial effects to health [44]. The use of alternative supplements, therefore, is unnecessary [5]. Our results were similar to previous studies, suggesting alternative supplement use is commonly reported by women [45].

### 4.5. Food Safety Practices

As seen in GUiNZ, a large percentage of women were aware of food safety recommendations [26]. There were, however, differences in foods avoided, with more women avoiding high-risk foods and beverages in this study. These results also showed higher adherence to food safety recommendations than was observed in South Auckland women in 2005 [46]. Alcohol was the most commonly avoided substance during both pregnancy and lactation, yet a number (8%) of women continued to consume alcohol during pregnancy. The consequences of alcohol consumption are widely understood and NZPBG recommends avoiding alcohol [5]. In this study, there were differences in alcohol consumption between women’s education level, with tertiary-educated women being two times more likely to avoid alcohol. These discrepancies between alcohol consumption suggest that further pregnancy-related alcohol campaigns are required, particularly for those with secondary-level education. The use of alcohol-related handouts was lower than for other topics. This could explain why some women continue consuming alcohol.

### 4.6. Dietary Information Sources

Women acquired dietary information from a range of sources that were not dissimilar to sources identified in GUiNZ [19]. Midwives were the most influential information source during both pregnancy and lactation. It seems that during pregnancy nutrition advice is commonly received from qualified health professionals, such as midwives. Dissemination of current evidence for dietary recommendations during pregnancy and lactation is a vital role that these health professionals provide; however, previous studies have reported that midwives lack knowledge around nutrition recommendations and do not feel confident giving nutrition advice, particularly for vegetarian women or those with health conditions [47,48].

As women’s dietary choices are often influenced by advice from midwives, further strategies to improve midwives’ knowledge or the increased availability of nutrition professionals would be warranted. Nearly the opposite was observed during lactation, with the majority of women relying on alternative health practitioners, the internet, and friends or family. The reasoning behind this difference in information procurement is likely because of the current NZPBG. The guidelines have a large emphasis on dietary recommendations during pregnancy, particularly food safety, but little on lactation [5]. Additionally, there is a change in health professionals available to women. LMCs will continue to care for women until six weeks postpartum, after which a Well-child provider will be available. Similar to midwives, previous research has indicated that Well-child care providers are not confident in providing nutrition advice and often nutrition education is not their key priority [49]. The lack of evidence-based support during lactation may be the reason for food removal trends. Information received from all sources was commonly about foods and beverages to avoid rather than what should be added or included in the diet. This was translated into more women removing and limiting than adding or increasing foods during pregnancy and lactation.

Women are recommended to increase their servings of food groups; however, practical explanations of how recommendations can be met are not given [5]. Instead, there is a large emphasis on the importance of removing specific high-risk foods such as alcohol and raw meats [5]. From previous studies, women found dietary recommendations easier to adhere to when foods were specifically mentioned [50]. Specifically mentioning the nutrients of concern, dietary requirements, and how to meet these requirements could be a way of improving the current recommendations. Nearly 60% of women already had children, which was a major factor for the limited use of antenatal classes. Women tended to attend antenatal classes during their first pregnancy only. Currently there are no regulations for the quantity or quality of nutrition advice given at antenatal classes, however, they may provide dietary advice [51]. Additionally, as attendance reduced during subsequent pregnancies, in this study the importance of other mediums for evidence-based dietary information, particularly if recommendations change, is emphasised.

This study provides valuable information surrounding the dietary choices of 458 geographically diverse pregnant and lactating NZ women. Previously in NZ there has been limited research investigating women’s dietary choices, food safety practices, and nutrition information sources, particularly during lactation. Our findings therefore provide important insight into what choices women are making and possible reasons why these choices are made. It also provides reasoning for why further research in this area is important to allow the best possible outcomes for NZ women and their offspring.

Although this study provides valuable findings it does also have limitations. The selected cohort’s demographics were predominantly NZ European, highly educated, food secure, and of good health status, thus not representative of the NZ population. This limitation reduces the ability to directly translate the research findings to improve the current New Zealand population-based strategies, and further research using a representative cohort is required. Our methods also included limitations. Collecting our data through online questionnaires, particularly those that were administered retrospectively and those that investigated dietary intake, may allow potential biased results secondary to participant self-selection, memory recall, food classification, question understanding, and truthfulness.

## 5. Conclusions

Women make dietary changes during pregnancy and lactation including adding, limiting, and avoiding foods. Dietary changes in pregnancy were influenced by many reliable information sources such as NZPBG, health professionals, or because of food safety concerns. In lactation, women more frequently received dietary advice from possibly less reliable sources, such as alternative health practitioners, the internet, and friends or family. Other common reasons for dietary change in lactation were because of concerns about the impact of the maternal diet on infant symptoms. The limited use of evidence-based information sources, such as health professionals and/or the NZPBG, in lactation highlights the need for changes in how information is communicated to women during this time. Food safety practices were generally followed; however, there is room for improvement.

## Figures and Tables

**Table 1:** Participant characteristics.

		Total *n* (%)
Participants		458 (100%)
Mean age (years ± SD)		32.5 ± 6
Ethnicity (Multiple responses allowed)	NZ European	433 (95%)
	Māori	42 (9%)
	Pacific Island	18 (4%)
	Asian	23 (5%)
	Other *	33 (7%)
Qualification	Secondary	77 (17%)
	Tertiary	380 (83%)
First baby	No	261 (57%)
Number of children	One	192 (74%)
	Two or more	68 (26%)
	Choose not to answer	1 (0%)
Diagnosis during pregnancy (multiple responses allowed)	Iron deficiency	219 (48%)
	Anaemia	29 (6%)
	Heartburn	160 (35%)
	Gestational diabetes	23 (5%)
	High blood pressure	17 (4%)
	Other ^#^	45 (10%)

* Other ethnicities: African, South African, British, Kurdish, Latin American, Russian, Scottish. ^#^ Other diagnoses: hyperemesis gravidarum, pre-eclampsia, cholestasis, fibroids, hypothyroidism, morning sickness, prolapsed disc, low platelets, tachycardia, constipation, polyhydramnios, B12 deficiency, thrush.

**Table 2:** Foods and beverages consumed daily during pregnancy and lactation.

Daily Food Group Consumption	Pregnancy *n* (%)	Lactation *n* (%)
**Dairy**		
Milk	428 (93%)	187 (64%)
Milk alternatives	73 (16%)	73 (25%)
Yoghurt	213 (47%)	100 (34%)
Cheese	116 (25%)	96 (33%)
Ice cream	34 (7%)	24 (8%)
**Lean meat, poultry, seafood, eggs, nuts, seeds, or legumes**		
Red meat	189 (41%)	151 (52%)
Poultry	230 (50%)	148 (51%)
Fish	23 (5%)	14 (5%)
Legumes	52 (11%)	41 (14%)
Nut/s or nut butters	211 (46%)	149 (51%)
Seeds	126 (28%)	92 (32%)
Tofu	8 (2%)	6 (2%)
**Breads and cereals**		
Bread	376 (82%)	245 (84%)
Breakfast cereals	273 (60%)	159 (55%)
**Fruit and vegetables**		
Fruit	404 (88%)	241 (83%)
Vegetables	410 (89%)	258 (89%)
**Beverages**		
Water	451 (98%)	283 (98%)
Tea	222 (48%)	136 (47%)
Coffee	193 (42%)	146 (50%)

**Table 3:** Foods and beverages added or increased in the diet during pregnancy and lactation.

Foods Added or Increased	Pregnancy *n* (%)	Lactation *n* (%)
Dairy products	211 (48%)	66 (23%)
Nuts	136 (31%)	75 (26%)
Green leafy vegetables	130 (29%)	68 (24%)
Meat	84 (19%)	39 (14%)
Salmon	56 (13%)	31 (11%)
Fortified cereals	58 (13%)	36 (13%)
No additions	139 (31%)	134 (47%)
Other *	42 (10%)	23 (8%)

* Pregnancy: spirulina, peanut butter, eggs, confectionary, celery, seeds, tomato, bread, fruit, jalapenos, iron rich foods, bran, Quorn, orange juice, white fish, porridge, seaweed, avocado, vegetables, fruit, herbal tea, olives, dates, tofu, sardines, water. * Lactation: oats, flaxseeds, alcohol, sushi, meal replacement drinks, brewer’s yeast, tofu, sardines, water.

**Table 4:** Foods and beverages avoided in the diet during pregnancy and lactation.

Foods and Beverages Avoided	Pregnancy *n* (%)	Lactation *n* (%)
Alcohol	408 (92%)	176 (62%)
Raw (unpasteurised) milk and milk products	379 (86%)	99 (35%)
Raw, smoked, or pre-cooked fish or seafood	371 (84%)	57 (20%)
Cold pre-cooked meats	335 (76%)	39 (14%)
Processed meats	315 (71%)	42 (15%)
Ready-made salads	313 (71%)	28 (10%)
Tahini	300 (68%)	29 (10%)
Foods containing raw egg	296 (67%)	24 (8%)
Soft-serve ice cream	288 (65%)	37 (13%)
Hummus	255 (58%)	16 (6%)
Soft pasteurised cheese	232 (52%)	35 (12%)
Cream or custard	212 (48%)	29 (10%)
No foods or beverages avoided	52 (12%)	84 (30%)
Other *	13 (3%)	12 (4%)

* Pregnancy: mayonnaise, sprouts, coffee, caffeine, eggs. * Lactation: caffeinated coffee, onion, spicy food, dairy products, chocolate.

**Table 5:** Foods limited in the diet during pregnancy and lactation.

Foods Limited in Diet	Pregnancy *n* (%)	Lactation *n* (%)
Canned fish	160 (36%)	36 (13%)
Bluff or Pacific oysters, or Queen scallops	136 (31%)	43 (15%)
Longer lived and larger fish	130 (29%)	30 (11%)
Deep sea or lake fish	128 (29%)	33 (12%)
Brown seaweed	128 (29%)	37 (13%)
Red or green seaweed	118 (27%)	39 (14%)
No limitations	85 (19%)	97 (34%)
Choose not to answer	17 (4%)	8 (3%)

**Table 6:** Reasoning for taking supplements in pregnancy and lactation.

Reasoning for Taking Supplements	Folic Acid Pregnancy *n* (%)	Folic Acid Lactation *n* (%)	Iodine Pregnancy *n* (%)	Iodine Lactation *n* (%)	Other Pregnancy *n* (%)	Other Lactation *n* (%)
Health professional’s advice	396 (93%)	39 (52%)	388 (92%)	144 (81%)	201 (65%)	84 (49%)
Following NZPBG	254 (60%)	14 (19%)	221 (53%)	36 (20%)	103 (33%)	20 (12%)
Advice from family member or friend	53 (12%)	6 (8%)	44 (10%)	4 (2%)	50 (16%)	20 (12%)
Advice from internet, magazine, book, or newspaper	37 (9%)	4 (5%)	27 (6%)	4 (2%)	27 (9%)	17 (10%)
Blood tests confirmed a deficiency	NA	NA	11 (3%)	2 (1%)	28 (9%)	5 (3%)
Other *	15 (4%)	27 (36%)	10 (2%)	26 (15%)	76 (25%)	74 (43%)

* Pregnancy: lacking in diet (vegan, vegetarian), reduce morning sickness, instead of Elevit, increases fertility, common knowledge, took in previous pregnancy, always taken, tiredness, boost nutrition, overall health and energy. * Lactation: lacking in diet (vegan, vegetarian) finishing off supplements, supporting milk supply, ensure dietary adequacy, post-partum haemorrhage, always taken, sleep, tiredness, boost nutrition, overall health and energy.

**Table 7:** Greatest influence on dietary choices.

Greatest Influence on Dietary Choices	Pregnancy *n* (%)	Lactation *n* (%)
Midwife	164 (37%)	86 (30%)
NZPBG	109 (25%)	30 (11%)
Family and friends	34 (8%)	35 (12%)
Internet	20 (5%)	23 (8%)
Obstetrician	22 (5%)	2 (1%)
GP	16 (4%)	8 (3%)
Books, magazines, and/or newspaper	6 (1%)	3 (1%)
Not sure	22 (5%)	13 (5%)
Alternative health practitioner	11 (2%)	74 (26%)
Other	38 (9%)	10 (4%)

* Pregnancy: antenatal classes, fast food advertisements, own professional background (GP or dietitian), own knowledge, no-one, appetite, common sense, dietitian/nutritionist, nausea, pamphlets, choose not to answer. * Lactation: antenatal classes, lactation consultant, fast food advertisements, own professional background (GP or dietitian), own knowledge, no advice received, appetite, common sense, dietitian/nutritionist, mixed sources, choose not to answer.

**Table 8:** Advice received from information sources in pregnancy and lactation.

Advice Received	Pregnancy			Lactation		
	LMC Advice *n* (%) *n* = 361	NZPBG *n* (%) *n* = 109	Antenatal Class *n* (%) *n* = 69	Midwife *n* (%) *n* = 86	Alternative Health Practitioner *n* (%) *n* = 74	Internet *n* (%) *n* = 23
Foods to consume	243 (67%)	75 (69%)	47 (68%)	58 (67%)	53 (71%)	15 (65%)
Beverages to consume	172 (48%)	61 (56%)	34 (49%)	43 (50%)	32 (43%)	8 (35%)
Foods to avoid/limit	321 (89%)	108 (99%)	59 (86%)	51 (59%)	37 (50%)	14 (61%)
Beverages to avoid/limit	270 (75%)	100 (92%)	61 (88%)	52 (60%)	26 (21%)	13 (57%)
How much food to consume	76 (21%)	23 (21%)	12 (17%)	21 (24%)	21 (29%)	5 (22%)
Supplements to take	258 (71%)	55 (50%)	17 (25%)	45 (52%)	53 (71%)	8 (35%)
Weight changes	160 (44%)	32 (29%)	19 (28%)	16 (19%)	0	9 (39%)
Importance of the diet during pregnancy or breastfeeding	158 (44%)	47 (43%)	34 (49%)	41 (48%)	26 (36%)	12 (52%)
Other *	11 (3%)	0	2 (3%)	2 (2%)	0	0

* Pregnancy: how to avoid Gestational diabetes, pamphlets, discouraged dieting, eat less fruit (too high in sugar), second pregnancy so did not discuss food in detail, morning sickness weight loss, drinks to lower blood pressure. * Lactation: baby symptoms, how to improve own diet to help baby, how to increase milk production.

**Table 9:** Dietary information sources used during lactation.

Lactation Information Sources	Total *n* (%)
Lead maternal carer	218 (77%)
Internet	190 (67%)
Family and/or friends	190 (67%)
NZPBG	129 (45%)
Plunket	125 (44%)
Other parents	124 (44%)
Health professional/s	93 (33%)
Books, magazines, and/or newspaper	87 (31%)
Television	8 (3%)
Radio	3 (1%)
Other *	13 (5%)

* Osteopath, nutritionist, Facebook, dietitian, lactation consultant, our Health Visitor, Pacifica nurses, handouts, apps, birth care.
